# Bending the curve: Modeling the impact of reducing risk factors for noncommunicable diseases to control future health expenditures in Latin America and the Caribbean

**DOI:** 10.1371/journal.pgph.0004791

**Published:** 2025-07-18

**Authors:** Andres I. Vecino-Ortiz, Timothy Roberton, Angelica López-Hernández, Caitlin M. Noonan, Angela P. Vega Landaeta, Daniel Maceira, Yvonne N. Flores, Claudio A. Mora-García, Paulina Giusti, T. Alafia Samuels, Natalia Palacio-Martínez, Andrea Prado, Carla Machado, Charmaine Metivier, Christine Laptiste, Althea La Foucade, Vyjanti Beharry, Krishna D. Rao

**Affiliations:** 1 Department of International Health, Health Systems program, Johns Hopkins Bloomberg School of Public Health, Baltimore, Maryland, United States of America; 2 Faculty of Health and Medical Sciences, School of Population and Global Health, University of Western Australia, Perth, Australia; 3 Pontificia Universidad Javeriana, Instituto de Salud Pública,; 4 University of Buenos Aires, Economics Department; National Council for Scientific and Technical Research (CONICET); Center for the Study of State and Society (CEDES), Buenos Aires, Argentina; 5 Unidad de Investigación Epidemiológica y en Servicios de Salud, Morelos, Instituto Mexicano del Seguro Social, Cuernavaca, Morelos, México; 6 UCLA Department of Health Policy and Management, Fielding School of Public Health, Los Angeles, CA, USA; 7 UCLA Center for Cancer Prevention and Control Research and UCLA-Kaiser Permanente Center for Health Equity, Fielding School of Public Health and Jonsson Comprehensive Cancer Center, Los Angeles, CA, USA; 8 Business School, INCAE University, San Jose, Costa Rica; 9 Instituto de Análisis y Gestión, Lima, Peru; 10 Caribbean Institute for Health Research, The University of the West Indies, Kingston, Jamaica; 11 Departamento Administrativo Nacional de Estadística, Bogota, Colombia; 12 School of Medicine, Universidade Federal de Minas Gerais, Belo Horizonte, Brazil; 13 HEU, Centre for Health Economics, The University of the West Indies, St. Augustine Campus, St. Augustine, Trinidad and Tobago; University of Modena and Reggio Emilia: Universita degli Studi di Modena e Reggio Emilia, ITALY

## Abstract

Addressing the World Health Organization’s noncommunicable disease (NCD) “best buys” is key to reducing the disease burden in Latin America and the Caribbean (LAC). Yet, the potential impact of addressing NCD risk factors on current health expenditures (CHE) in LAC countries is unknown. This study uses both Global Burden of Disease (GBD) data and administrative information to model the impact of addressing four risk factors on CHE trends for 24 LAC countries. A comparative risk assessment model estimates changes in CHE associated with reducing five NCDs. Reducing the prevalence of the four risk factors by 10% could save $ 185 billion in cumulative expenditure by 2050 (1.32% of cumulative expenditure from 2020 to 2050) for all LAC countries assessed, with substantial heterogeneity across risk factors. Reducing the prevalence of high blood pressure had the largest impact. On average, a reduction of 10% in high blood pressure, tobacco use, high blood glucose, and alcohol use would reduce cumulative CHE by US$59bn (0.4% of the cumulative CHE by 2050), US$68bn (0.5%), US$46bn (0.3%), and US$12bn (0.1%), respectively for all LAC countries. While addressing NCD risk factors is a key step to improving health in LAC countries, the impact on CHE is relatively small though meaningful in absolute terms, and additional strategies need to be implemented to control increasing CHE levels that threaten health systems’ sustainability.

## Introduction

In most Latin American and Caribbean (LAC) countries, health expenditure growth is a cause for concern due to the increasing demographic, epidemiological, and technological pressures that health systems face in a context of growing levels of debt, insufficient fiscal revenues, and high out-of-pocket payments [1–3], which have worsened after facing the economic and social consequences of the COVID-19 pandemic [4,5]. Health expenditure growth represents a significant sustainability risk for health systems as it directly threatens the objective of achieving universal health coverage in the context of constrained resources.

Specifically, the financial burden of noncommunicable diseases (NCD) in Latin American and Caribbean (LAC) countries is rising. Only cardiovascular disease represented costs of nearly US$30 billion in LAC countries in 2010 [6,7]. Since there are specific risk factors associated with NCDs, it is reasonable to assess whether addressing the main NCD risk factors can have an actual impact on the financial burden of NCDs. Previous research has attempted to establish the attributable effects of the prevalence of risk factors and diseases on health expenditures. For example, a study in China found that changes in risk factors and disease prevalence determined health expenditure growth [8]. Similar results were found in Australia, where the attributable effect of aging on CHE growth is mainly related to comorbidities in the Australian population [9].

However, the literature available to measure the impact of NCDs and their associated risk factors on future health spending remains sparse, mostly focused on country-level studies estimating catastrophic and out-of-pocket health expenditures [10–13], but rarely focusing on current health expenditures (CHE) [1–3]. For example, Haakenstad et al. [14] used the World Health Surveys to estimate the impact of disease prevalence on catastrophic health expenditure. They found that a 10% increase in the prevalence of cardiovascular disease is related to a 1.5% to 1.9% increase in cardiovascular-specific CHE. Diving into the catastrophic health expenditure literature, the same Disease Control Priorities in its third edition identified that the four conditions listed in the WHO “best buys” are responsible for 3 out of 4 deaths and most of the growth on catastrophic health expenditures in LMIC’s [15–18].

Considering the gap in the literature assessing the effect of modifying the prevalence of risk factors on current health expenditure growth, the aim of this study is to estimate such effect in 24 LAC countries.

## Methods

In this study, we simulated the potential impact that changes in the prevalence of four different risk factors in LAC countries between 2020 and 2050 would have on current health expenditures linked to the burden of five main NCDs.

### Risk factors

The four behavioral risk factors were originally defined as those belonging to the four WHO “best buys”: tobacco use, harmful alcohol use, unhealthy diet, and physical inactivity. However, while these four risk factors are also present in the Global Burden of Disease study (GBD), unfortunately, unhealthy diet and physical inactivity are variables with known comparability issues [19]. We replaced these with two alternative metabolic risk factors as defined by GBD 2019 [20]; high blood glucose and high systolic blood pressure, because they are biological determinants of the leading causes of death: heart attack and stroke.

### Outcomes

The conditions selected for this study also come from the WHO “Best Buys”: cardiovascular disease, chronic respiratory disease (specifically chronic obstructive pulmonary disease), diabetes mellitus type 2, and cancer. All four conditions are caused by the WHO Best Buys risk factors and similarly correspond to the risk factors selected in this study. In addition to those four conditions, we included chronic kidney failure due to its relationship to the aforementioned risk factors and the high cost that its treatment represents [21,22], for a total of five main outcomes measured.

### Data

We used data to establish 1) baseline levels of CHE by ICD-10 chapter; 2) prevalence of the four selected risk factors and the five selected conditions; 3) the population attributable fraction (PAF) of each condition caused by each risk factor. Finally, we obtained data on the share of the costs attributable to each condition within each ICD-10 chapter. More details about how the different pieces of data match will be explained later in this section.

In this study, we used data from three main sources: 1) GBD 2019 data [23] was used to obtain a) the prevalence of the four selected risk factors and the five selected conditions mentioned above, and b) to map the PAF of the selected four risk factors onto the prevalence of the five selected conditions [24]. 2) We used country-level estimated of 2020 CHE and projections through 2050 from Rao et al [7]. 3) Having country-specific prevalence of the five selected conditions and CHE at the ICD-10 level, we needed to capture the magnitude of CHE related to each of the five selected conditions within the respective ICD-10 chapter.

Given the difficulty to obtain CHE data at this level of granularity, we took advantage of data from the Colombian Ministry of Health and Social Protection information system (known as RIPS) that provides CHE by condition to estimate the fraction of the costs attributable to each of the five selected conditions within their respective ICD-10 codes [25]. What this implies is that we had to assume that the distribution of costs of, for example, chronic kidney failure within the XIV ICD-10 code (*Diseases of the genitourinary system*) is similar across countries. It is key to emphasize that we are not assuming CHE attributable to chronic kidney failure is similar in all countries but that rather, the share of this condition within the respective ICD-10 chapter is the same. This is a necessary assumption to conduct this study, recognizing that all health systems are different, and those differences explain the changes in the cost structure across the board. Fortunately, the differences in the share of costs of any condition within its respective ICD-10 chapter is substantially less likely. The granularity of data available in our seven index countries only allows us to perform this specific estimation (distribution of expenditures of each condition within the respective ICD-10 chapter) at a national level in Colombia. Even though most Latin American and Caribbean countries share similar epidemiology in the relative prevalence of noncommunicable diseases [23], we hope better data collection in the future allows researchers to refine these estimations for every country. This assumption allows us to obtain a plausible value between the minimum possible value (zero) and the maximum possible value (the total cost of the respective ICD-10 chapter). The RIPS database is the most complete database available in the country, and the only one we found available with this level of granularity at a national level.

### GBD 2019 mapping to ICD-10 chapters

For changes in prevalence, we used GBD 2019 data [23], available from the Institute for Health Metrics and Evaluation (IHME) [24]. We excluded GBD 2021 because of potential distortions arising from the COVID-19 pandemic, which affected access and diagnosis of noncommunicable conditions. The causes of GBD are classified into four levels. Level 1 has three large cause groupings: communicable, maternal, neonatal, and nutritional deficiencies; non-communicable diseases; and injuries. At level 2, there are 21 disease and injury categories –the closest classification to the 22 ICD-10 Chapters. The finest level of detail in causes is provided at levels 3 and 4. For example, level 3 includes specific causes, e.g., cerebral infarction, and level 4 provides more detailed information, e.g., cerebral infarction due to thrombosis. This is the most detailed classification for disease causes in the GBD, corresponding to blocks A00 to Z99 in the ICD-10. In the latter example, for the ICD-10, this would correspond to I63 (level 3 GBD) and I63.01 (level 4 of GBD) In this study, we included all changes in prevalence from the GBD2019 study for all Level 2 and Level 3 disease categories for all five-year age groups from 0 to 85 years between 1990–2019. Most Level 2 disease categories are mapped into a single ICD chapter, and we used ICD Level 3 categories for diseases in Chapters IV, VII, VIII, and XIV [26]. This approach meant we had meaningful data for 21 of 22 ICD-10 Chapters, except for Chapter XXI, which includes occupational exposures, incarcerated individuals, dependence on enabling machines, pre-employment examinations, medical certificates, immunization, and screening procedures not elsewhere classified. Thus, for ICD Chapter XXI, we assumed a conservative flat prevalence trend.

### Baseline current health expenditure trends

We used CHE projections from Rao et al. [7], as our default scenario. Briefly, current health expenditure (CHE) in LAC countries were based on estimated changes in three underlying factors: prevalence change of ICD-10-chapter distribution, population change (aging), and economic growth for seven countries. The residual parameter from these three factors represents other important factors affecting cost growth, such as technological change, changes in wages for healthcare workers beyond growth, policy changes improving coverage and quality, etc. To extrapolate from the seven index countries (Argentina, Brazil, Colombia, Costa Rica, Mexico, Peru, and Trinidad and Tobago) to others in the region, LAC countries were grouped into four groups based on 2015–2019 average per capita health expenditures using age-expenditure profiles. A statistical deterministic model to forecast CHE towards 2050 was developed including the change of population size, the relative change of prevalence of disease, a composite term that captures the annual real growth in CHE due to income growth and other factors, and CHE in the previous year for each disease and age group. Rao’s study broadly shows that per capita CHE will increase across the LAC region and that economic growth and technology will be the main drivers of health expenditure growth in the LAC region.

### Comparative risk assessment (CRA) model

Comparative risk assessment (CRA) is a well-known strategy to conduct ex-ante modeling and has been widely used in previous studies [27–29].

Following mapping the GBD 2019 categories to ICD-10 codes, we used the GBD 2019 estimates of the PAF of the four selected risk factors to the five selected diseases in terms of disability-adjusted life years (DALYs).

Next, each of the five selected conditions was located within an ICD-10 chapter, thanks to the mapping described above. For example, our selected condition, “cardiovascular disease”, is within chapter IX “Diseases of the circulatory system”, or cancer corresponds to the entire chapter II, “Neoplasms”. A similar process was done with the other three conditions. Since the baseline CHE trends are available by ICD-10 chapter, it was necessary for us to understand the percentage of CHE that each of the selected conditions represents within its respective ICD-10 chapter. As explained earlier, only one country provided data with such granularity, the RIPS data from Colombia. We estimated the 2015–2019 average share of CHE for each condition within the respective ICD-10 chapter and assumed that this value applies for all 24 countries studied.

In Table 1, we display the average share of CHE for each selected condition within its respective ICD-10 code by country, based on Colombia’s RIPS data.

**Table 1:** Average share of current health expenditures within its respective ICD-10 Chapter in Colombia (2015-2019).

Condition assessed	ICD-10 chapter	Percentage of ICD-10 chapter expenditures attributable to each condition within its respective chapter+
Cancer	II	100.00
Diabetes mellitus type 2	IV	24.85
Cardiovascular disease (CVD)	IX	91.91
Chronic Obstructive Pulmonary Disease (COPD)	X	36.89
Chronic kidney failure	XIV	37.2

+This share was used to extrapolate costs of each condition within the respective ICD-10 chapter.

**Fig 1:**
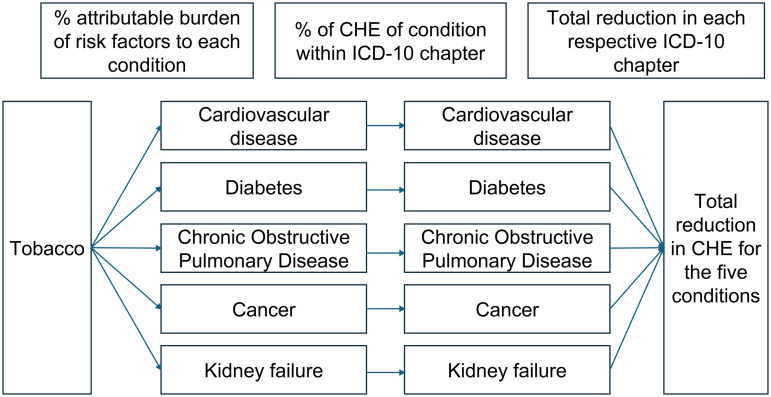
Example of comparative risk assessment model exemplifying the estimation of the effect of a 10% reduction in the prevalence of tobacco use on total current health expenditures. *This figure represents the potential policy impact described in Eq. 1. Each risk factor (tobacco exposure in this case) can potentially have effects in some, or all of the five conditions measured. These conditions represent a share of health expenditures in each of their respective ICD-10 chapters. These data is obtained from Colombia’s RIPS dataset and is assumed to be similar in other countries. In turn, each ICD-10 chapter represents a share of total health expenditures, which can vary by country depending on epidemiological conditions.


In order to carry out the CRA, we calculated the expected impact on health expenditures by multiplying 1) the projected CHE for any given year for each country, age profile, and ICD-10 Chapter; 2) the share of CHE attributed to each of the five selected conditions within their respective ICD-10 Chapter; 3) the percent change in the prevalence of each risk factor modeled reductions of 5%, 10%, and 25%; 4) the PAF expressed as a DALYs ratio. We assume that a change in a risk factor is linearly translated into changes in the prevalence of the corresponding condition after ten years. For this reason, we interpolated the final effects over a ten-year period in a linear fashion. See Fig 1 and equation 1 for further clarity.

$$P_{ijkvpt+10}={CHE}_{ijvt}\;x\;{ShareCol}_{jkv}\;x\;{ChangePrevRF}_{pt}\;x\;\frac{{DALY}_{ijkvpt}}{{DALY}_{ijkvt}}\;$$ (Eq 1)

Where the expected policy impact on current health expenditures *P* in country *i*, age category *j*, outcome *k*, risk factor *p*, and year *t* is represented by the projected CHE for each country i, age category j, ICD-10 code *v*, and year t multiplied by: (i) the share of the age-specific expenditures (ShareCol) that each outcome represents within its respective ICD-10 chapter *v* in Colombia (averaged between 2015 and 2019); (ii) the deterministic scenarios (5%, 10%, 25%) in the prevalence of the risk factor (ChangePrevRF); and (iii) the share of DALYs attributable to each risk factor for each condition. The choice of scenarios (5%, 10%, and 25%) was made discretionally, considering potential scenarios of change in risk factor prevalence. Since we need to obtain DALYs lost associated to changes in the prevalence of each of the risk factors in each country, we also present those results. All impacts are then projected through 2050 with a 3% discount rate as done in previous work [30]. Readers must be aware that since we are addressing a set of the main NCD risk factors and the main NCD conditions, we might not be capturing the full extent of the effect of reducing the risk facts on expenditures. However, we believe that by addressing the most important conditions associated with them the degree of potential underestimation is relatively low.

All the point estimates presented here are deterministic in nature and therefore, we have not produced confidence intervals around them. The data analyses and projections were done using Stata and JavaScript, respectively.

## Results

In order to estimate the potential impact of changes in the prevalence of risk factors on current health expenditures, we carried out a comparative risk assessment model where we examined three scenarios of prevalence reduction in the four selected risk factors on total CHE and DALYs, specifically through changes in the five selected conditions compared to projected counterfactual CHE values [7].

### Savings in terms of future health expenditures

In Table 2, we present cumulative savings with discounted values for costs through 2050 by reducing risk factors by 10% (second scenario). The discounted values were estimated at a 3% annual discount rate. In S1 Appendix, we display the estimates for all LAC countries for reductions of 5%, 10% and 25% for all risk factors.

**Table 2:** Discounted cumulative savings by 2050 due to 10% reduction in selected risk factors, by country (2018 million US$, and percentage of base-case cumulative expenditure).

	Tobacco 10%		Hypertension 10%		High blood glucose 10%		Alcohol 10%		Savings from 10% reduction in all four risk factors	
**Latin American countries**										
Argentina	6,191	0.42%	6,639	0.45%	3,984	0.27%	811	0.06%	17,626	1.20%
Bolivia	159	0.15%	389	0.38%	313	0.30%	17	0.02%	877	0.85%
Brazil	36,375	0.61%	22,756	0.38%	13,901	0.23%	8,938	0.15%	81,970	1.38%
Chile	4,029	0.38%	6,997	0.65%	4,120	0.39%	657	0.06%	15,803	1.48%
Colombia	2,178	0.22%	6,267	0.63%	4,514	0.45%	164	0.02%	13,123	1.32%
Costa Rica	390	0.23%	962	0.56%	598	0.35%	30	0.02%	1,981	1.15%
Ecuador	653	0.19%	1,555	0.46%	1,342	0.39%	54	0.02%	3,604	1.06%
El Salvador	113	0.19%	338	0.57%	261	0.44%	8	0.01%	719	1.21%
Guatemala	296	0.17%	884	0.49%	702	0.39%	24	0.01%	1,907	1.06%
Honduras	176	0.25%	334	0.48%	266	0.38%	26	0.04%	803	1.16%
Mexico	4,376	0.21%	11,936	0.58%	10,142	0.49%	566	0.03%	27,020	1.31%
Nicaragua	110	0.23%	274	0.56%	200	0.41%	13	0.03%	597	1.23%
Panama	493	0.19%	1,514	0.58%	1,119	0.43%	99	0.04%	3,225	1.24%
Paraguay	325	0.32%	497	0.49%	333	0.33%	42	0.04%	1,198	1.17%
Peru	863	0.16%	3,432	0.62%	1,915	0.35%	118	0.02%	6,329	1.14%
Uruguay	868	0.48%	954	0.53%	531	0.29%	110	0.06%	2,462	1.36%
All Latin American countries	57,596	0.42%	65,728	0.48%	44,242	0.32%	11,678	0.09%	179,243	1.32%
**Caribbean countries**										
Bahamas	39	0.19%	134	0.63%	77	0.36%	14	0.07%	264	1.25%
Barbados	15	0.18%	59	0.68%	43	0.49%	7	0.08%	124	1.44%
Belize	10	0.26%	20	0.50%	13	0.33%	2	0.05%	46	1.14%
Dominican Republic	928	0.34%	1,764	0.64%	975	0.35%	110	0.04%	3,777	1.37%
Guyana	12	0.18%	31	0.47%	25	0.38%	3	0.04%	71	1.08%
Jamaica	84	0.33%	151	0.58%	116	0.45%	11	0.04%	362	1.40%
Suriname	24	0.27%	44	0.49%	36	0.41%	3	0.04%	107	1.21%
Trinidad and Tobago	199	0.35%	471	0.83%	331	0.58%	33	0.06%	1,033	1.82%
All Caribbean countries	1,313	0.32%	2,673	0.66%	1,616	0.40%	183	0.04%	5,784	1.42%
**All countries**	**58,908**	**0.42%**	**68,401**	**0.49%**	**45,857**	**0.33%**	**11,861**	**0.08%**	**185,027**	**1.32%**

Among the four risk factors, the largest CHE reductions would be achieved by reducing prevalence of hypertension, except in Brazil, where tobacco reduction would bring the largest reductions.

Reducing the prevalence of risk factors would result in significant cumulative savings over the long term. These savings are driven by the relative reduction in CHE for each scenario and the absolute CHE for each country. Note that these reductions in CHE should be viewed as ‘gross reductions’ because there will likely be investments made to achieve changes in risk factors.

### Savings in terms of DALYs

In addition to reductions in health expenditures, the risk factor interventions examined would also result in health gains that are not directly monetized in this research study but that any reader can estimate using standard estimates on the value of statistical life. Table 3 shows the estimated reduction in DALYs for the four risk factors in three different scenarios (reduction of 5%, 10% and 25% of the baseline prevalence of the risk factor). Across the entire region, a 10% reduction in the prevalence of high blood glucose and hypertension would reduce DALYs lost associated with the five conditions by 2.8% and 2.6%, respectively. Results on the effect of the reduction of risk factors on DALYs by level of reduction and country can be found in S2 Appendix.

**Table 3:** Average percentage reduction in DALYs among all LAC countries, by prevalence reduction scenario and condition.

	Tobacco			Hypertension			High blood glucose			Alcohol		
	5%	10%	25%	5%	10%	25%	5%	10%	25%	5%	10%	25%
Cardiovascular diseases	0.74	1.47	3.68	2.62	5.23	13.08	1.15	2.30	5.76	0.08	0.17	0.42
Cancer (neoplasms)	0.78	1.57	3.92				0.20	0.39	0.98	0.20	0.41	1.02
Chronic obstructive pulmonary disease	2.29	4.58	11.45									
Diabetes mellitus type 2	0.64	1.29	3.22				5.00	10.00	25.01			
Chronic kidney disease				2.82	5.64	14.10	1.55	3.10	7.74			

## Discussion

To our knowledge, this is among the first studies to model the impact of addressing risk factors on current health expenditures. We present the results of a model that links reductions in the prevalence of four selected risk factors to the reduction in CHE attributed to five selected conditions in the hopes that these data contributes to the decision-making process in LAC countries when it comes to the growth of health expenditures.

This study shows that by addressing the four selected risk factors, countries can expect long-term CHE and DALY reductions. The magnitudes of those savings are substantial, reaching cumulative savings of around 185 billion USD by 2050 (in 2018 USD) for all 24 LAC countries included, equivalent to 1.32% of cumulative expenditure between 2019–2050. However, those impacts might not be as high, relative to the expected CHE growth as one might expect, and their effects might range greatly depending on the actual risk factor being addressed, the level of reduction, and the country.

Importantly, DALY reductions generally exceed CHE savings, suggesting economic benefits beyond cost savings are likely given that economic gains can be derived from higher productivity resulting from reducing the burden of disease of these conditions through different policy tools such as health taxes targeting tobacco, alcohol, and some food products.

This study allows us to differentiate between the actual reductions in the burden of disease attributable to the risk factors and the final reductions in CHE, where the latter are affected by the former, but also modified by the relative weight that every condition has within the respective ICD-10 chapter, the weight of the respective chapter in CHE in the country and other modifying factors such as the cost structure of the health system or country-specific medical inflation.

Generally, addressing hypertension reduced CHE by a larger magnitude than high blood glucose, which in turn, reduced CHE by a larger magnitude than tobacco. This is possibly related to the recent rise in hypertension and metabolic diseases in the region compared to tobacco use [31,32]. Yet, the magnitude of these effects is not negligible. Most countries experience continued CHE growth larger than GDP growth, and the compounded effects of CHE reductions over time associated with either reducing risk factor prevalence or controlling costs are counted in the millions to the billions of USD (at 2018 prices). Modest but statistically significant reductions have also been found in previous research when the prevalence of risk factors contributes with between 3 and 5% of expenditure growth [8,9]. Importantly, we observed important regional differences on the impact of risk factors between Latin American and Caribbean countries, such as high blood pressure, likely due to differences in disease burden and treatment patterns.

Most of the countries have an adult tobacco smoking prevalence higher than 10%. In the case of high blood pressure, we note that the association between reduction in disease burden and CHE seems less linear but substantial. Hypertension is the most important risk factor, of those assessed, driving health expenditures, due to the high prevalence of the condition and the costs of its complications [33]. It is noticeable that the variation in the disease burden is larger than that of CHE, perhaps implying that resource utilization and prices are more relevant drivers of CHE than disease prevalence. In the case of high blood glucose, which is substantially higher in Caribbean countries and Mexico, there appears to be a clear relationship between the reduction in disease burden and CHE, even though changes in disease burden are associated with relatively much smaller changes in CHE. Regarding alcohol consumption, this risk factor seems to have a more pronounced effect on DALYs than on health expenditures. A potential reason for this is the important effect that alcohol has on injuries and external causes of burden of disease, many of them leading to a higher number of deaths that might be better captured in the DALY data than in the health expenditure data, which only include expenditures associated to the five conditions mentioned. This becomes more evident among the countries with some of the highest consumption levels in the region (Argentina, Paraguay and Brazil) [34]. The case of Brazil is interesting because of the significantly higher effect on health expenditures with respect to other countries. Despite Brazil has a high prevalence of alcohol consumption in patterns of “binge drinking” and other studies have identified large costs attributed to alcohol consumption, the reason for the deviation with respect to other countries in the region cannot be concluded from the data and warrants further exploration [35,36].

While we have compared the relative economic and health gains that could be achieved by reducing the prevalence of each selected risk factor, we emphasize that the *feasibility* of achieving the same prevalence reduction in each risk factor is likely different. For example, while a 10% reduction in tobacco may not bring the same gains as a 10% reduction in hypertension, it may be more feasible in the short term through the implementation of known tobacco reduction strategies such as taxation compared to improving hypertension screening and treatment. Thus, it might represent a more effective policy option even if it yields a smaller impact. These determinations will be highly country-specific and involve other factors beyond the scope of this study and we encourage researchers and decision-makers to explore the trade-offs between feasibility and impact further.

It is important to note that while this is a novel study, such novelty comes with limitations that must be considered. First, we are using models that estimate counterfactuals based on secondary and modeled data, such as GBD 2019, and therefore they are susceptible to the assumptions and the data that feed the model. Also, the data we use to estimate the share of expenditures of each condition within its respective ICD-10 chapter is assumed to be similar across all countries. However, it is key to highlight that there are no previous attempts to estimate the value of addressing the main risk factors in LAC, and these figures will provide relevant inputs to decision-makers to make better-informed decisions and to collect better and more data so future studies can be conducted without the need of relying on these assumptions.

Second, our results might yield underestimated values for two main reasons. First, we only captured the effect of four selected risk factors on five selected conditions, and the effect of those risk factors on other conditions is not captured. Second, we are only estimating direct costs incurred by the health system when treating these conditions, and therefore, the indirect costs associated with the disease burden, such as human capital losses, are not captured here. Therefore, this study displays a lower (conservative) bound for both CHE and overall costs derived from these risk factors. While we believe that by addressing the most important conditions associated with those risk factors, the degree of potential underestimation of direct costs is relatively low, we must enphasize that this study takes the perspective of the health system, as opposed as the perspective of the society, and therefore it is not accounting for the indirect costs (e.g., human capital losses) of these risk factors.

Third, implementation and feasibility matter. Two of the selected risk factors can be addressed mainly through primary prevention, tobacco or alcohol taxation, smoking cessation programs, etc. For the other risk factors (high blood pressure and high blood glucose), secondary prevention strategies are likely more feasible when looking at health sector policies. Limitations such as limited primary health care systems, health worker capacity, health systems resiliency, screening, or treatment availability are important and might require larger initial investments with long-term observable goals that often times are less appealing to elected officials. We have estimated the implications of risk factor reductions in prevalence, acknowledging that it is easier to reduce some of them and harder to reduce some others. In this work, we don’t have the ability to estimate the costs of implementing these interventions, but certainly more work is needed on that front in order to inform decision-makers about a comprehensive estimation of the full benefit of WHO best buys, potentially expanding on previous efforts [15].

Fourth, the reductions in CHE do not take into account upfront investment costs, which will likely be needed to achieve the changes in risk factors mentioned here. This health care expenditure forecasting model does not account for the money spent up front to implement interventions that reduce risk factors. This work serves as an input for future cost-benefit analysis addressing these risk factors. At the same time, reductions in DALYs might increase productivity and human capital, yielding economic gains that are not captured in this study.

Fifth, the mapping of GBD cases to ICD-10 level 2 categories is imperfect and misclassification is always possible.

Sixth, we are using country-specific data for global burden of disease from the burden of disease study and ICD-10 chapter country-specific estimated health care cost data from Rao et al [7]. However, the participation, in terms of costs, of the specific condition (diabetes mellitus type 2, cardiovascular disease, COPD, and chronic kidney disease) within the respective ICD-10 chapter (IV, IX, X, XIV) is extrapolated from data from Colombia, given the impossibility to get more granular data form other countries. The extrapolation of the data from one country might introduce biases in some specific countries where the epidemiology is different, so for example costs associated to these risk factors through cardiovascular disease might be overestimated in Bolivia given that this country has a higher prevalence of Chagas disease than Colombia and therefore, a larger share of the costs estimated for chapter IV can be attributable to Chagas disease instead of any of the four risk factors assessed. Similarly, COPD might be related to tobacco in most countries but in Peru costs for chapter X might be more strongly associated to indoor cooking than in Colombia, leading to potential overestimation of the attributable burden. Naturally, this problem would not happen with cancer as we are estimating the entire cost of the ICD-10 chapter (chapter II in the case of cancer). We acknowledge that using Colombian data to extrapolate the share of the costs within ICD-10 chapters might be an important limitation for some conditions and some countries and we don’t have record of any other data exercise that would require this type of assumption. However, this is an important assumption to estimate the overall effects of risk factors on specific conditions rather than on entire ICD-10 chapters. As mentioned earlier, these are assumptions needed given the novelty of the type of study. We are confident that while in some specific cases, as described, the results might be overestimated, generally, most of these risk factors represent a similar burden in most countries considering their similar epidemiology of disease. Health systems across the region are different, and that might change their costs, but it might be less likely that the health system structure changes the distribution of costs of any condition within the respective ICD-10 code. The need for this assumption highlights the relevance of more granular ICD-10 three-digit expenditure data in LAC countries, so future research does not need to assume health expenditure distributions of an ICD-10 three-digit code within a given chapter.

Seventh, we assume a 10-year linear lag on the reduction of CHE attributable to the change in the risk factor. Since population-level disease progression might not always be linear, this approach might not consider cumulative exposure, policy implementation, and health system responsiveness. For example, a tobacco reduction program might not fully translate into changes in behavior linearly, and the effects of such a program on lung cancer and related costs might be relatively low during the first decade. However, we use the linear lag for all conditions because using a different lag for every combination of disease and risk factor would make it an unfeasible exercise.

Eighth, this work was done before the COVID-19 pandemic, and it is possible that the pandemic may have affected the estimated trends in risk factor exposure and healthcare utilization. We encourage more research and future calibration of these results in the coming years.

The absolute savings are substantial (US$184.72 billion), ranging between 0.03 and 2.31 percent reduction in CHE, though modest relative to projected CHE growth because CHE increases in the intensive margin (more intensive and costly technology for a given disease) rather than on the extensive margin (increasing overall coverage for previous uncovered conditions), implying that larger savings come from cost control measures than from reducing the burden of disease [7,8].

Our study shows that while reducing NCD risk factors is key to increasing human capital and promoting healthier and happier societies; unfortunately, those reductions do not automatically imply a large effect on CHE growth.

## Supporting information

S1 Appendix Percentage change in future health expenditures by 2050 associated to reductions of the prevalence of the four selected risk factors, compared to the compared to the expected trend in risk factor prevalence. (DOCX)

S2 Appendix Percentage reduction in 2050 DALYs from 5 causes, by scenario and country. (DOCX)
