# Supplementary material for: Bending the curve: Modeling the impact of reducing risk factors for noncommunicable diseases to control future health expenditures in Latin America and the Caribbean
Source: PLOS Glob Public Health. 2025 Jul 18;5(7):e0004791. doi: 10.1371/journal.pgph.0004791 (PMC12273971; doi:10.1371/journal.pgph.0004791)
Supplement: S1 Appendix — (DOCX) [file pgph.0004791.s001.docx]

S1 Appendix. Percentage change in future health expenditures by 2050 associated to reductions of the prevalence of the four selected risk factors, compared to the compared to the expected trend in risk factor prevalence.

Latin America.

|  | **Tobacco** | | | **Hypertension** | | | **High blood glucose** | | | **Alcohol** | | |
| --- | --- | --- | --- | --- | --- | --- | --- | --- | --- | --- | --- | --- |
|  | **5%** | **10%** | **25%** | **5%** | **10%** | **25%** | **5%** | **10%** | **25%** | **5%** | **10%** | **25%** |
| Argentina | 0.32 | 0.64 | 1.59 | 0.33 | 0.66 | 1.65 | 0.20 | 0.41 | 1.02 | 0.04 | 0.08 | 0.20 |
| Bolivia | 0.13 | 0.25 | 0.64 | 0.34 | 0.69 | 1.71 | 0.26 | 0.51 | 1.28 | 0.01 | 0.02 | 0.04 |
| Brazil | 0.44 | 0.88 | 2.19 | 0.29 | 0.57 | 1.43 | 0.18 | 0.35 | 0.88 | 0.10 | 0.21 | 0.52 |
| Chile | 0.25 | 0.50 | 1.25 | 0.46 | 0.91 | 2.28 | 0.27 | 0.54 | 1.35 | 0.04 | 0.08 | 0.20 |
| Colombia | 0.15 | 0.31 | 0.77 | 0.46 | 0.92 | 2.30 | 0.33 | 0.66 | 1.65 | 0.01 | 0.02 | 0.06 |
| Costa Rica | 0.17 | 0.33 | 0.83 | 0.42 | 0.83 | 2.09 | 0.26 | 0.53 | 1.32 | 0.01 | 0.02 | 0.06 |
| Ecuador | 0.14 | 0.28 | 0.70 | 0.34 | 0.68 | 1.71 | 0.30 | 0.61 | 1.51 | 0.01 | 0.02 | 0.05 |
| El Salvador | 0.15 | 0.29 | 0.73 | 0.46 | 0.92 | 2.30 | 0.35 | 0.70 | 1.76 | 0.01 | 0.02 | 0.05 |
| Guatemala | 0.13 | 0.26 | 0.64 | 0.39 | 0.78 | 1.94 | 0.31 | 0.62 | 1.55 | 0.01 | 0.02 | 0.05 |
| Honduras | 0.21 | 0.42 | 1.06 | 0.41 | 0.82 | 2.04 | 0.32 | 0.63 | 1.59 | 0.03 | 0.06 | 0.14 |
| Mexico | 0.15 | 0.31 | 0.77 | 0.43 | 0.87 | 2.17 | 0.37 | 0.74 | 1.85 | 0.02 | 0.04 | 0.10 |
| Nicaragua | 0.18 | 0.36 | 0.91 | 0.45 | 0.90 | 2.26 | 0.33 | 0.65 | 1.63 | 0.02 | 0.04 | 0.09 |
| Panama | 0.13 | 0.25 | 0.63 | 0.40 | 0.80 | 2.01 | 0.30 | 0.60 | 1.49 | 0.02 | 0.05 | 0.12 |
| Paraguay | 0.24 | 0.48 | 1.21 | 0.37 | 0.74 | 1.85 | 0.25 | 0.50 | 1.26 | 0.03 | 0.06 | 0.15 |
| Peru | 0.11 | 0.22 | 0.55 | 0.45 | 0.90 | 2.24 | 0.24 | 0.48 | 1.20 | 0.01 | 0.02 | 0.06 |
| Uruguay | 0.34 | 0.67 | 1.68 | 0.36 | 0.72 | 1.80 | 0.21 | 0.42 | 1.04 | 0.04 | 0.08 | 0.21 |
