# Supplementary material for: Bending the curve: Modeling the impact of reducing risk factors for noncommunicable diseases to control future health expenditures in Latin America and the Caribbean
Source: PLOS Glob Public Health. 2025 Jul 18;5(7):e0004791. doi: 10.1371/journal.pgph.0004791 (PMC12273971; doi:10.1371/journal.pgph.0004791)
Supplement: S2 Appendix — (DOCX) [file pgph.0004791.s002.docx]

S2 Appendix. Percentage reduction in 2050 DALYs from 5 causes, by scenario and country.

**Latin America.**

|  | **Tobacco** | | | **Hypertension** | | | **High blood glucose** | | | **Alcohol** | | |
| --- | --- | --- | --- | --- | --- | --- | --- | --- | --- | --- | --- | --- |
|  | **5%** | **10%** | **25%** | **5%** | **10%** | **25%** | **5%** | **10%** | **25%** | **5%** | **10%** | **25%** |
| Argentina | 1.15 | 2.30 | 5.76 | 1.15 | 2.31 | 5.77 | 0.96 | 1.93 | 4.82 | 0.17 | 0.34 | 0.85 |
| Bolivia | 0.43 | 0.85 | 2.13 | 1.00 | 2.01 | 5.02 | 1.22 | 2.43 | 6.09 | 0.02 | 0.04 | 0.11 |
| Brazil | 0.98 | 1.95 | 4.89 | 1.36 | 2.72 | 6.79 | 1.19 | 2.39 | 5.97 | 0.11 | 0.22 | 0.56 |
| Chile | 0.83 | 1.66 | 4.14 | 1.35 | 2.70 | 6.74 | 1.17 | 2.34 | 5.86 | 0.15 | 0.30 | 0.76 |
| Colombia | 0.62 | 1.23 | 3.08 | 1.19 | 2.38 | 5.96 | 1.34 | 2.69 | 6.72 | 0.04 | 0.07 | 0.18 |
| Costa Rica | 0.67 | 1.33 | 3.33 | 1.42 | 2.85 | 7.12 | 1.27 | 2.55 | 6.37 | 0.06 | 0.12 | 0.30 |
| Ecuador | 0.45 | 0.89 | 2.23 | 1.10 | 2.20 | 5.50 | 1.39 | 2.78 | 6.95 | 0.04 | 0.08 | 0.21 |
| El Salvador | 0.41 | 0.81 | 2.03 | 1.45 | 2.90 | 7.25 | 1.72 | 3.44 | 8.59 | 0.02 | 0.03 | 0.08 |
| Guatemala | 0.43 | 0.86 | 2.15 | 1.22 | 2.43 | 6.08 | 1.92 | 3.84 | 9.59 | 0.02 | 0.04 | 0.11 |
| Honduras | 0.76 | 1.52 | 3.80 | 1.55 | 3.10 | 7.74 | 1.43 | 2.86 | 7.16 | 0.09 | 0.18 | 0.44 |
| Mexico | 0.57 | 1.15 | 2.87 | 1.30 | 2.59 | 6.49 | 1.96 | 3.91 | 9.79 | 0.06 | 0.11 | 0.28 |
| Nicaragua | 0.51 | 1.02 | 2.55 | 1.60 | 3.20 | 8.00 | 1.74 | 3.49 | 8.72 | 0.05 | 0.10 | 0.25 |
| Panama | 0.47 | 0.94 | 2.36 | 1.27 | 2.55 | 6.37 | 1.63 | 3.26 | 8.14 | 0.09 | 0.19 | 0.47 |
| Paraguay | 1.01 | 2.02 | 5.04 | 1.43 | 2.85 | 7.13 | 1.45 | 2.89 | 7.23 | 0.14 | 0.29 | 0.71 |
| Peru | 0.28 | 0.55 | 1.38 | 1.11 | 2.22 | 5.56 | 1.01 | 2.01 | 5.04 | 0.04 | 0.08 | 0.19 |
| Uruguay | 1.22 | 2.44 | 6.09 | 1.06 | 2.11 | 5.28 | 0.76 | 1.52 | 3.80 | 0.18 | 0.36 | 0.90 |

**Caribbean.**

|  | **Tobacco** | | | **Hypertension** | | | **High blood glucose** | | | **Alcohol** | | |
| --- | --- | --- | --- | --- | --- | --- | --- | --- | --- | --- | --- | --- |
|  | **5%** | **10%** | **25%** | **5%** | **10%** | **25%** | **5%** | **10%** | **25%** | **5%** | **10%** | **25%** |
| Bahamas | 0.47 | 0.95 | 2.37 | 1.57 | 3.14 | 7.84 | 1.36 | 2.72 | 6.80 | 0.15 | 0.29 | 0.73 |
| Barbados | 0.37 | 0.74 | 1.84 | 1.18 | 2.37 | 5.92 | 1.95 | 3.89 | 9.73 | 0.14 | 0.28 | 0.69 |
| Belize | 0.67 | 1.34 | 3.35 | 1.30 | 2.61 | 6.52 | 1.56 | 3.13 | 7.82 | 0.12 | 0.23 | 0.59 |
| Dominican Republic | 0.91 | 1.83 | 4.57 | 1.68 | 3.35 | 8.38 | 1.14 | 2.28 | 5.70 | 0.11 | 0.21 | 0.54 |
| Guyana | 0.57 | 1.13 | 2.83 | 1.66 | 3.32 | 8.31 | 1.86 | 3.72 | 9.30 | 0.14 | 0.27 | 0.68 |
| Jamaica | 0.70 | 1.41 | 3.52 | 1.21 | 2.41 | 6.03 | 1.79 | 3.58 | 8.95 | 0.08 | 0.15 | 0.38 |
| Suriname | 0.89 | 1.77 | 4.44 | 1.37 | 2.75 | 6.87 | 1.75 | 3.51 | 8.77 | 0.11 | 0.22 | 0.55 |
| Trinidad and Tobago | 0.70 | 1.40 | 3.50 | 1.44 | 2.88 | 7.19 | 2.17 | 4.34 | 10.86 | 0.06 | 0.12 | 0.31 |
